# Bivariate analysis of barley scald resistance with relative maturity reveals a new major QTL on chromosome 3H

**DOI:** 10.1038/s41598-019-56742-y

**Published:** 2019-12-30

**Authors:** Xuechen Zhang, Ben Ovenden, Beverley A. Orchard, Meixue Zhou, Robert F. Park, Davinder Singh, Andrew Milgate

**Affiliations:** 1 NSW Department of Primary Industries, Wagga Wagga Agricultural Institute, Wagga Wagga, NSW 2650 Australia; 2 Tasmanian Institute of Agriculture, University of Tasmania, Private Bag 1375, Prospect, TAS 7250 Australia; 3 Plant Breeding Institute, The University of Sydney, Cobbitty, Private Bag 4011, Narellan, NSW 2567 Australia

**Keywords:** Plant breeding, Biotic

## Abstract

The disease scald of barley is caused by the pathogen *Rhynchosporium commune* and can cause up to 30–40% yield loss in susceptible cultivars. In this study, the Australian barley cultivar ‘Yerong’ was demonstrated to have resistance that differed from Turk (*Rrs1* (Rh3 type)) based on seedling tests with 11 *R. commune* isolates. A doubled haploid population with 177 lines derived from a cross between ‘Yerong’ and the susceptible Australian cultivar ‘Franklin’ was used to identify quantitative trait loci (QTL) for scald resistance. A QTL on chromosome 3H was identified with large effect, consistent with a major gene conferring scald resistance at the seedling stage. Under field conditions, a bivariate analysis was used to model scald percentage of infected leaf area and relative maturity, the residuals from the regression were used as our phenotype for QTL analysis. This analysis identified one major QTL on chromosome 3H, which mapped to the same position as the QTL at seedling stage. The identified QTL on 3H is proposed to be different from the *Rrs1* on the basis of seedling resistance against different *R. commune* isolates and physical map position. This study increases the current understanding of scald resistance and identifies genetic material possessing QTLs useful for the marker-assisted selection of scald resistance in barley breeding programs.

## Introduction

Scald is a serious foliar disease in barley (Hordeum vulgare) that is caused by *Rhynchosporium commune*. The pathogen can cause up to 30–40% yield loss in susceptible cultivars and is found in all barley-growing regions worldwide^1^. Control of scald disease requires a multi-facetted approach, including application of fungicides, cultural disease management, manipulation of sowing date and the use of resistant cultivars^2^. *R. commune* populations have changed rapidly in response to newly-developed fungicides and resistant plant cultivars^3–6^. One of the most sustainable strategies for *R. commune* management is to develop and deploy disease-resistant barley cultivars through the introgression and pyramiding of different resistance genes (major or minor). Traditional methods of phenotypic selection for complex patho-systems such as scald on barley can be improved through detailed genetic studies, which allow the implementation of marker-assisted selection (MAS) in breeding programs.

Scald resistance is governed by both major and minor genes. Major resistance genes provide high levels of resistance at all plant growth stages, while minor resistance genes generally provide partial levels of resistance at the adult plant stage^7,8^. Reduced scald symptoms in adult stage plants under field conditions might also result from disease escape through physical barriers to infection^9^. In terms of scald resistance in barley, flowering time, plant height and canopy structure can affect scald symptoms by physically limiting the upward spread of the splash-dispersed pathogen^8^. The major scald resistance genes discovered so far have been mainly identified through experiments with seedlings via inoculation with specific isolates^8^. The problem with using major scald resistance genes in breeding programs is a lack of durability. Quantitative genes are thought to be more durable, and it has been suggested that pyramiding these genes could reduce the ability of *R. communes* to rapidly acquire new virulence combinations^10^.

A large number of QTLs for scald resistance have been discovered in barley. Two genomic regions in particular have been frequently associated with resistance; the *Rrs1* locus on chromosome 3H and the *Rrs2* locus on chromosome 7H^8,11^. These two loci have been detected in different mapping populations, across different environments, under glasshouse conditions after inoculation with specific isolates, and under field conditions^8,11^. Progress of our understanding of these loci has been made through fine mapping studies^12,13^. However, it remains unknown if the QTLs detected at each of these two loci are alleles of the same gene, or if they are part of a closely linked gene cluster at each locus^14,15^. For the *Rrs1* locus, multiple major and minor scald resistance alleles or loci have been identified, supporting its importance in barley germplasm worldwide^12,16–21^. Bjornstad, *et al*.^16^ suggests that previously identified scald resistance loci Rh, Rh1, Rh3, Rh4 and Rh7 are all alleles of the *Rrs1* locus. A single dominant gene *Rrs1* (Rh type) was first identified in cultivar ‘Brier’^22^. *Rrs1* (Rh4 type) was identified in cultivars ‘La Mesita’, ‘Trebi’ and ‘Osiris’, with a similar allele, termed Rh4^2^, identified in the cultivar ‘Modoc’^23^. In ‘Turk’ and ‘Atlas46’, another resistance allele *Rrs1* (Rh3 type) was identified closely linked to the *Rrs1* (Rh4 type) from ‘Modoc’^23^. *Rrs1* (Rh type) was the first scald resistance locus reported in barley^23^ with an associated RFLP marker of cMWG680, positioned at 455.3 Mb on chromosome 3H on the pseudomolecules Morex V 2.0 2019^19^. The location of the *Rrs1* (Rh4 type) locus was further fine mapped to an interval of less than 9 Mb at 448.4 Mb on the pseudomolecules Morex V 2.0 2019^24^ and 490.0 Mb on the pseudomolecules Morex 2017^11,12^ from Spanish barley landraces.

In this study, we performed screening against 11 *R. commune* isolates at seedling stage to demonstrate that resistance in the cultivar ‘Turk’ (a widely used international source of *Rrs1* (Rh3 type)) differs from that resistance in the cultivar ‘Yerong’. These results contradicted previous findings that suggested ‘Yerong’ carries a scald resistance QTL at the *Rrs1* locus on chromosome 3H^25^. To further investigate this finding, screening of a ‘Yerong’/‘Franklin’ doubled haploid (DH) population was conducted for seedling resistance against four *R. commune* isolates and adult plant resistance under natural field conditions across three years. To overcome potential confounding effects of differences in plant growth stage and the onset of flowering (determined as relative maturity) on scald resistance QTL detection within the population, a linear mixed model with a bivariate approach was used to analyse the field data and derive a resistance phenotype from the linear relationship between relative maturity and percentage of infected leaf area. Using this approach, a major QTL on chromosome 3H from Yerong was detected for scald resistance at both seedling and adult plant stage. The QTL detected did not map to the *Rrs1* locus, suggesting the presence of a new and useful scald resistance QTL in ‘Yerong’.

## Results

### Differential cultivar screening

The cultivar ‘Atlas46’ (*Rrs1 + Rrs2*) was resistant to all the isolates used in this study, with BLUP scores under 2.3, except isolate WAI2840, which was virulent on all cultivars with a BLUP score of more than 3.5 (Table 1). ‘Turk’ (*Rrs1*) was resistant to all the isolates used in this study with scores under 2.4, except two isolates WAI2439 and WAI2840. Cultivars carrying *Rrs2* (‘Atlas’ and ‘Atlas46’) were resistant against isolate WAI2439 (Table 1). Cultivars conferring *Rrs1* (‘Turk’ and ‘Atlas46’) showed higher level of resistance than ‘Atlas’ and ‘Yerong’ against two isolates, WAI1245 and WAI2464. Cultivars carrying either *Rrs1* or *Rrs2* were resistant against isolates WAI453, WAI2466, WAI2471 and WAI2636.

**Table 1:** Predicted values (BLUPs) with 95% confidence intervals for seedling resistance of different barley cultivars against 11 different *R. commune* isolates.

Cultivar	Resistance	WAI453	WAI1245	WAI2439	WAI2463	WAI2464	WAI2466	WAI2470	WAI2471	WAI2473	WAI2636	WAI2840
‘Atlas’	*Rrs2*	2.4 ± 0.8	3.3 ± 0.9	2.3 ± 0.8	3.1 ± 0.8	4.0 ± 0.9	2.4 ± 0.6	2.8 ± 0.8	2.2 ± 0.8	2.9 ± 1.2	2.5 ± 0.5	3.5 ± 0.9
‘Atlas46’	*Rrs1*(Rh3 type)* + Rrs2*	2.1 ± 0.8	2.0 ± 0.8	2.3 ± 0.8	2.2 ± 0.8	2.1 ± 0.9	1.9 ± 0.6	2.1 ± 0.9	2.0 ± 0.8	2.0 ± 0.8	1.8 ± 0.5	3.5 ± 0.8
‘Franklin’		4.2 ± 0.8	4.3 ± 0.8	3.5 ± 0.8	4.4 ± 0.8	4.4 ± 0.8	4.7 ± 0.5	4.4 ± 0.8	3.4 ± 1.3	3.6 ± 0.8	4.3 ± 0.5	4.4 ± 0.8
‘Litmus’	Susceptible	4.5 ± 0.8	4.4 ± 0.8	4.4 ± 0.9	4.5 ± 0.8	4.5 ± 0.8	5.2 ± 0.5	4.4 ± 0.8	4.0 ± 0.8	4.3 ± 0.8	4.1 ± 0.5	3.9 ± 1.2
‘Turk’	*Rrs1* (Rh3 type)	2.0 ± 0.8	2.2 ± 0.8	3.8 ± 0.8	2.4 ± 0.8	2.0 ± 0.8	1.7 ± 0.5	2.0 ± 0.8	2.0 ± 0.8	2.0 ± 0.8	1.7 ± 0.5	4.1 ± 0.8
‘Yerong’		3.1 ± 0.8	3.4 ± 0.8	3.2 ± 0.8	4.3 ± 0.8	4.3 ± 0.8	4.7 ± 0.5	4.3 ± 0.8	2.3 ± 0.8	3.2 ± 0.8	3.8 ± 0.5	4.3 ± 0.8

‘Turk’ was considerably more resistant than ‘Yerong’ against five isolates and equivalent against the remaining six isolates tested. Together, these results suggested that the *Rrs1* (Rh3 type) resistance possessed by ‘Turk’ was not present in ‘Yerong’. Compared to ‘Yerong’, ‘Atlas’ showed a higher level of resistance against isolates WAI2439, WAI2463, WAI2466, WAI2470 and WAI2636, suggesting *Rrs2* is absent in ‘Yerong’. ‘Yerong’ was resistant against WAI2471, and displayed moderate resistance against isolates WAI453, WAI1245, WAI2439 and WAI2473 with the scores between 3.0 and 3.4. ‘Yerong’ showed better resistance than ‘Franklin’ against isolates WAI453, WAI1245 and WAI2471with the overall disease scores of ‘Yerong’ being lower than those of ‘Franklin’. The cultivar ‘Litmus’ was susceptible to all the isolates in this study.

### QTLs for scald resistance at the seedling stage

**Figure 1:**
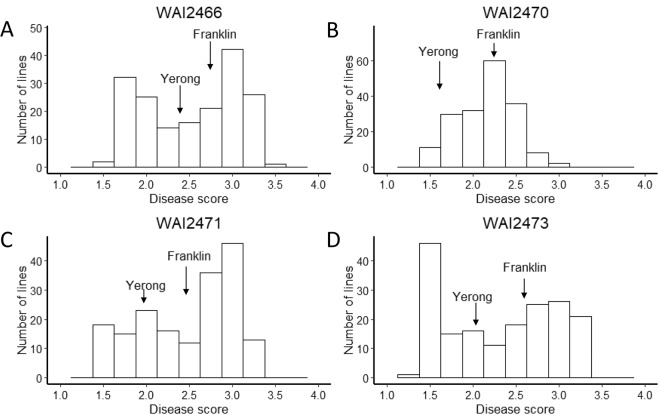
Frequency distribution of scald resistance to four pathogen isolates in the Yerong/Franklin population at the seedling stage different isolates. (**A**) WAI2466 (**B**) WAI2470 (**C**) WAI2471 (**D**) WAI2473. The positions of text labels “Yerong” and “Franklin” in the figure are based on the disease score of each parent at the seedling stage.

There were no significant differences (using 95% confidence intervals) in resistance to the four isolates WAI2466, WAI2470, WAI2471 and WAI2473 among the two parent cultivars in the seedling stage screening experiments. However, there was phenotypic variation in resistance to the different isolates among the DH population lines. The disease scores among DH population lines against WAI2466 showed a bimodal distribution of scores between resistant and susceptible lines (Fig. 1A). The variation of disease scores against WAI2470 was the lowest among all four isolates (Fig. 1B). The distribution of disease scores against WAI2471 was skewed towards higher levels of disease (Fig. 1C). In contrast, the distribution of disease scores against WAI2473 was skewed towards the lower end of the scoring scale (Fig. 1D).

**Figure 2:**
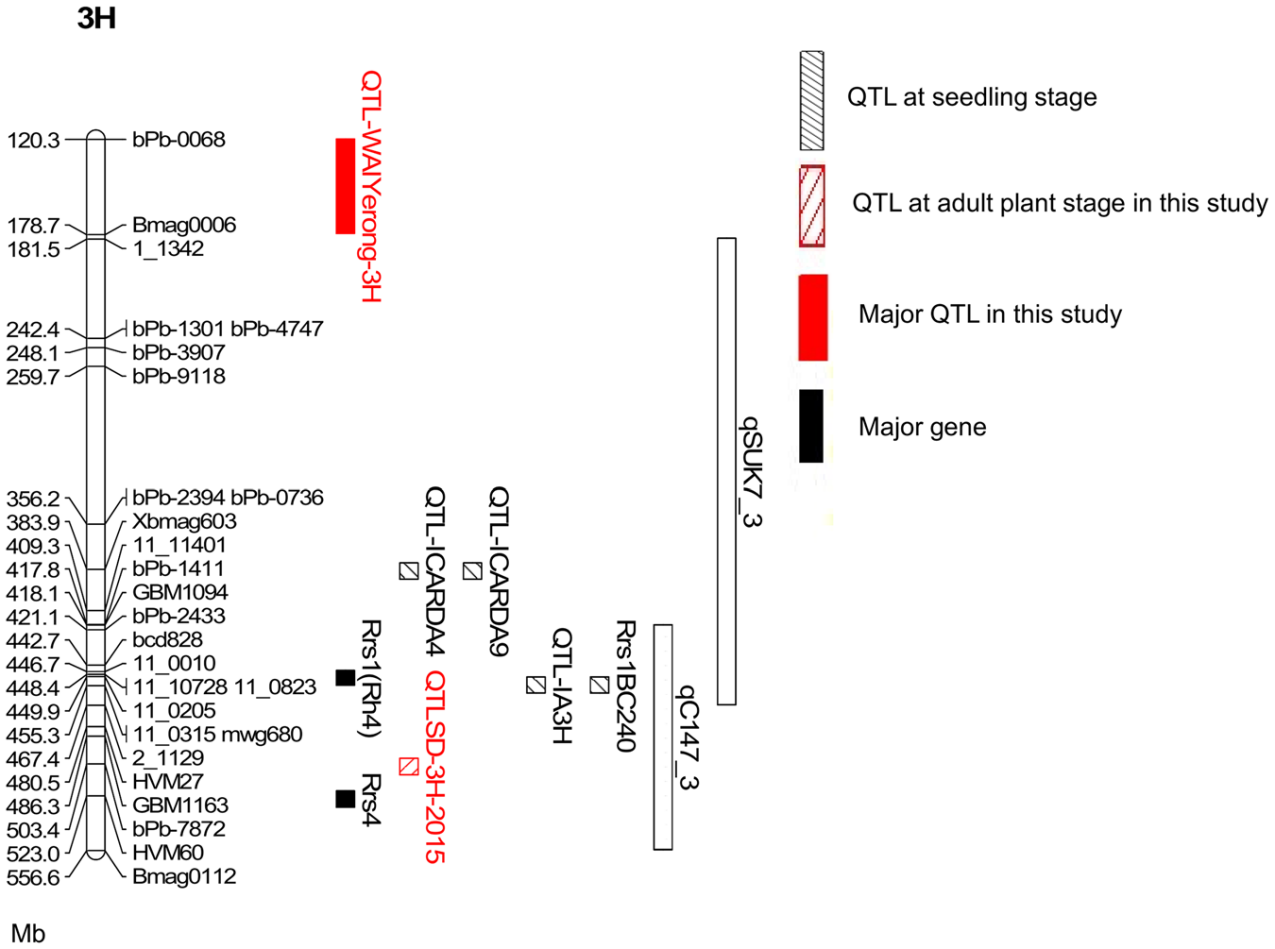
Positions of marker sequences on 3H pseudomolecule (100–600 Mb) pseudomolecules Morex V 2.0 2019. Flanking markers of the major QTL on chromosome 3H from ‘Yerong’ identified in this study (bPb-0068 and Bmag0006) mapped at 120.3 and 178.7 Mb on the physical map. Flanking markers of QTLD-3H-2015, bPb-7872 and bPb-8410 mapped at 486.2 and 503.4 Mb on the physical map. The physical position of fine mapped *Rrs1* (Rh4 type) is identified using flanking markers, at 448.4 Mb^12^. The original RFLP marker mwg680, which is closely linked to the *Rrs1* locus, is located at 455.3 Mb^19^. The resistance QTLs from ‘ICARDA4’ and ‘ICARDA9’ are mapped at 383.9 Mb based on marker Xbmag603^18^. The resistance QTL Rrs1BC240from wild barley H. *spontaneum* ‘CPI 109853’ is mapped at 455.3 Mb^18^. The locations of resistance QTLs identified from ‘Steptoe’ (qSUK7_3) and ‘CIho 3515’ (qC147_3) are cited from Coulter, *et al*.^17^. The resistance QTL QTL-IA3H from ‘Abyssinian’ is mapped at 455.3 Mb against four isolates^20^. Major resistance gene *Rrs4* is mapped at 523.0 Mb based on marker HVM60^52^.

One major QTL on chromosome 3H, designated as QTL-WAIYerong-3H, was identified for seedling resistance to all four different *R. commune* isolates (WAI2466, WAI2470, WAI2471 and WAI2473) at the same position (Table 2) at 178.7 Mb. Flanking markers of this QTL, bPb-0068 and Bmag0006, are mapped at 120.3 and 178.7 Mb on the pseudomolecules Morex V 2.0 2019 (Fig. 2). The LOD scores varied from 23.8 to 38.7, explaining more than 46% of the phenotypic variation (Table 2).

**Table 2:** Summary of QTLs for scald resistance at the seedling stage to four different isolates: WAI2466, WAI2470, WAI2471 and WAI2473, and Summary of the QTLs for all traits measured in the field experiments. QTLRM is the QTL for relative maturity, QTLSP is the QTL for scald percentage, and QTLD is the QTL for DRSRRM.

Trait	QTL name	Year	Chromosome	Linkage map position	Closest marker	LOD	R^2^	Additive effect
WAI2466-seedling	QTL-WAIYerong-3H		3H	54.1	Bmag0006	38.7	63.5	−0.43
WAI2470-seedling	QTL-WAIYerong-3H		3H	54.1	Bmag0006	23.8	46.2	−0.22
WAI2471-seedling	QTL-WAIYerong-3H		3H	54.1	Bmag0006	32.3	56.8	−0.41
WAI2473-seedling	QTL-WAIYerong-3H		3H	54.1	Bmag0006	38.4	63.1	−0.52
Plant height	QTLPH	2017	3H	124.1	bPb-7335	5.7	14.1	3.07
Relative maturity	QTLRM-2H.1-2015	2015	2H	31.3	bPb-4523	11.5	19.0	3.03
	QTLRM-7H-2015		7H	41.7	bPb-9601	8.2	12.9	−2.42
	QTLRM-2H.2-2015		2H	73.1	Bmac0093	7.6	11.7	2.43
	QTLRM-5H-2015		5H	65.1	bPb-7015	6.4	9.7	2.04
	QTLRM-2H.1-2016	2016	2H	18.1	bPb-3186	10.4	15.0	2.15
	QTLRM-7H-2016		7H	41.7	bPb-9601	9.9	14.1	−2.11
	QTLRM-5H-2016		5H	58.9	bPb-9476	8.0	11.2	1.88
	QTLRM-2H.2-2016		2H	73.1	Bmac0093	4.0	5.3	1.31
	QTLRM-2H.1-2017	2017	2H	31.3	bPb-4523	8.6	14.3	2.19
	QTLRM-7H-2017		7H	41.7	bPb-9601	8.1	13.5	−2.04
	QTLRM-2H.2-2017		2H	71.6	bPb-6881	5.2	8.3	1.75
	QTLRM-5H-2017		5H	65.1	bPb-7015	5.1	8.1	1.57
Scald percentage	QTLSP-3H-2015	2015	3H	69.6	bPb-7872	4.3	11.1	−4.80
	QTLSP-2H-2015		2H	31.3	bPb-4523	3.9	10.2	4.47
	QTLSP-3H-2016	2016	3H	54.1	Bmag0006	10.8	17.8	−4.18
	QTLSP-6H-2016		6H	142.3	bPb-3780	5.2	7.9	2.80
	QTLSP-2H-2016		2H	31.3	bPb-4523	5.2	7.9	2.76
	QTLSP-7H-2016		7H	53.0	bPb-5091	4.9	7.4	−2.68
	QTLSP-5H-2016		5H	124.8	bPb-0171	3.3	4.9	2.15
	QTLSP-7H-2017	2017	7H	68.5	bPb-4541	5.9	13.0	−4.53
	QTLSP-3H-2017		3H	54.1	Bmag0006	4.4	9.6	−3.87
DRSRRM	QTLD-3H-2015	2015	3H	69.6	bPb-7872	5.1	14.8	−4.83
	QTLD-3H-2016	2016	3H	54.1	Bmag0006	8.8	20.7	−3.76
	QTLD-7H-2017	2017	7H	68.5	bPb-4541	5.8	12.9	−4.49
	QTLD-3H-2017		3H	54.1	Bmag0006	4.4	9.7	−3.86

### Scald resistance under field conditions

**Figure 3:**
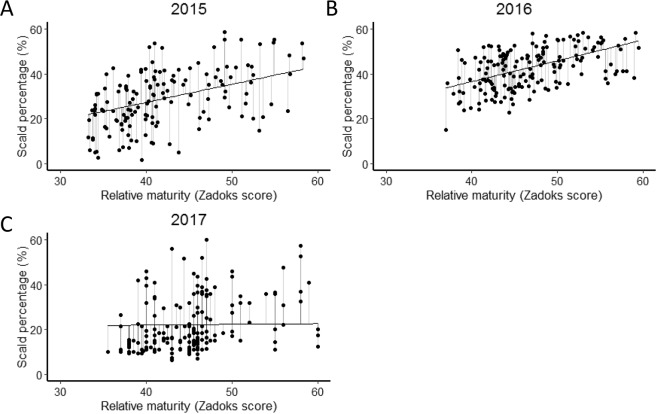
The scald percentage of infected leaf area in the DH population of ‘Yerong’/‘Franklin’ plotted against relative maturity (Zadoks score) across three years. (**A**) 2015 (**B**) 2016 (**C**) 2017.

The parent cultivars ‘Franklin’ and ‘Yerong’ showed similar levels of scald resistance and relative maturity across the different years (Table 3). There were no significant differences between ‘Franklin’ and ‘Yerong’ for any of the traits measured across the different years (p > 0.05). However, the DH lines showed substantial phenotypic variation in scald resistance and relative maturity (Fig. 3). Average disease incidence was more severe in 2016, reaching 43.0%, higher than the average scald percentages in 2015 (29.0%) and 2017 (22.1%) (Table 3).

**Table 3:** Phenotypic predicted values (BLUPs) of traits measured in the Yerong/Franklin population under field conditions.

Trait	Year	Average	Max	Min	Franklin	Yerong
Relative maturity	2015	42.1	58.4	33.3	38.2	38.3
	2016	47.0	59.5	37.0	42.8	45.9
	2017	45.2	61.0	35.5	43.0	41.5
Scald percentage	2015	29.0	63.4	−0.3	23.7	21.7
	2016	43.0	63.5	15.1	31.6	34.3
	2017	22.1	61.5	6.5	16.0	18.5
DRSRRM	2015	−0.1	34.8	−26.4	−2.2	−4.3
	2016	−0.2	19.7	−18.6	−7.6	−7.8
	2017	−0.1	39.3	−15.6	−6.1	−3.6
Plant height	2017	96.3	115.5	72.0	107.0	106.0

A significant correlation (Pearson’s correlation coefficient) between scald percentage of infected leaf area and relative maturity was observed among the DH lines (Fig. 3A) in 2015 (r = 0.50, p < 0.01), 2016 (r = 0.51, p < 0.01) (Fig. 3B). Later flowering lines (with lower relative maturity scores) tended to have a lower scald percentage. In 2017, although Pearson correlation coefficient between relative maturity and scald percentage of infected leaf area was significant (r = 0.34, p < 0.01), no correlation was indicated between relative maturity and scald percentage of infected leaf area from bivariate analysis (Fig. 3C). A phenotype value was calculated to account for the relationship between scald percentage and relative maturity by determining the residual values for scald percentage from a simple linear model of scald percentage and relative maturity. We termed this phenotype the deviation from the regression of scald percentage on relative maturity (DRSRRM) and used this as a phenotype for QTL analysis in our field trials.

### QTLs for relative maturity and plant height

**Figure 4:**
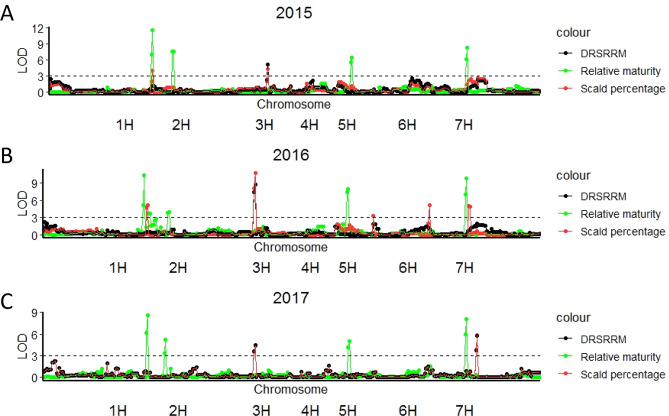
LOD values of QTLs detected for relative maturity, scald percentage and DRSRRM by MQM mapping across three years field experiments in the ‘Yerong’/‘Franklin’ DH population, years are presented separately as (**A**) 2015 (**B**) 2016 (**C**) 2017.The LOD values of each marker were plotted against the chromosomes.

Four QTLs for relative maturity (QTLRM) were identified in the Yerong/Franklin population consistently across all three years (Fig. 4 and Table 2): two on chromosome 2H, one on chromosome 5H and another one on chromosome 7H. These four QTLs explained more than 40% of the total phenotypic variance for relative maturity. One QTL for plant height (QTLPH) in 2017 was identified on chromosome 3H that explained 14.1% of the total phenotypic variance, with a LOD value of 5.7 (Table 2).

### QTL for scald resistance in the field

In 2015, one QTL for scald percentage (QTLSP) on chromosome 2H (QTLSP-2H-2015) was located at the same position as the 2H QTL for relative maturity (QTLRM-2H-2015) (Fig. 4A and Table 2). In 2016, QTLSP-2H-2016 and QTLRM-2H-2016 were also mapped at the same position on chromosome 2H (Fig. 4B and Table 2). Markers associated with QTLSP-7H-2016 and QTLRM-7H-2016 were also located close to each other (Fig. 4B and Table 2). QTLs for scald percentage were not mapped at the same position as QTLs for relative maturity in 2017 (Fig. 4C and Table 2).

The QTLs for DRSRRM (QTLD) in 2016 (QTLD-3H-2016) and 2017 (QTLD-3H-2017) were located at 178.7 Mb in the same position as QTL-WAIYerong-3H, and the resistant allele was also derived from the ‘Yerong’ parent. The QTL for DRSRRM (QTL-WAIYerong-3H) explained 20.7% of the phenotypic variance in 2016 (LOD value 8.8; Fig. 4 and Table 2). In 2015, the QTL for DRSRRM on chromosome 3H (QTLD-3H-2015) was 15 cM away from this major QTL on the linkage map, at 503.4 Mb on the pseudomolecules Morex V 2.0 2019. This QTL was still more than 50 Mb away from *Rrs1* (Rh4 type), between *Rrs1* (Rh4 type) (448.4 Mb) and *Rrs4* (523.0 Mb) on chromosome 3H, explaining 14.8% of phenotypic variance. QTLs were identified for DRSRRM on chromosome 3H across the three years at the same location as the QTLs detected for scald percentage. In 2017, a novel QTL for scald resistance (both scald percentage and DRSRRM) was identified on chromosome 7H from ‘Franklin’, locating at 69.9 Mb on the pseudomolecules Morex V 2.0 2019. This QTL explained 12.9% phenotypic variance with a LOD value of 5.8.

## Discussion

### Seedling stage scald resistance

Differential screening conducted in this study showed that the combination of major resistance loci *Rrs1* and *Rrs2* in the cultivar ‘Atlas46’ provides resistance to 10 out of 11 *R. commune* isolates from southern NSW. These results confirmed the effectiveness of pyramiding major resistance genes into one cultivar in providing broad protection against *R. commune*^7,26^. However, resistance gene combinations need to be found that do not rapidly select for the corresponding virulence gene combinations in the pathogen population. This remains a challenge with only a limited understanding of the ‘gene for gene’ interaction between *R. commune* and its host. We have identified specific *R. commune* isolates from NSW to distinguish the presence of *Rrs1* or *Rrs2*, comparable to the results of Hofmann, *et al*.^12^ and Wallwork and Grcic^7^. While isolates such as WAI2840 posed a threat to reliance on just these two major loci, they are valuable in detecting sources of seedling resistance other than *Rrs1* and *Rrs2* for use in resistance breeding.

Five of the *R. commune* isolates screened in this study were avirulent against ‘Turk’ and virulent against ‘Yerong’, indicating that the *Rrs1* (Rh3 type) allele from ‘Turk’ is not present in ‘Yerong’. ‘Yerong’ was resistant to isolate WAI2471, and had moderate levels of resistance against WAI453, WAI1245, WAI2439 and WAI2473 (Table 1). Further QTL analysis located a major QTL for scald resistance against four different isolates at the seedling stage to chromosome 3H, which was derived from ‘Yerong’. This QTL-WAIYerong-3H explained more than 46% of the phenotypic variation for scald resistance and is located at the same position of a QTL (QSc.YeFr-3H) identified using adult plant data in a previous study from the same DH population in Tasmania, Australia^25^. However, Li and Zhou (2011) were unable to distinguish this QTL from ‘Yerong’ from the *Rrs1 *locus.

The complete reference barley genome sequence enabled projection of QTLs from different studies onto two current versions of the barley physical map, the pseudomolecules Morex V 2.0 2019 and the pseudomolecules Morex 2017 (Supplementary Table S1)^11,24,27^. In our study, flanking markers of the QTL-WAIYerong-3H, bPb-0068 and Bmag0006, are located at 120.3 and 178.7 Mb respectively on the pseudomolecules Morex V 2.0 2019. The position is some distance away from previously fine mapped *Rrs1* (Rh4 type) which is located at a position of 448.4 Mb on chromosome 3H (Fig. 2)^12^. This also suggested the scald resistance QTL-WAIYerong-3H is different from *Rrs1*. Multiple major and minor scald resistance genes or QTLs have been identified on chromosome 3H, close to the *Rrs1* locus^12,16–20^. Among these QTL, the QTL-IA3H from cultivar ‘Abyssinian’ was identified in studies of seedling resistance to four *R. commune* isolates^20^. A QTL Rrs1BC240 for *R. commune* resistance from wild barley *H. spontaneum* was also identified at the position of *Rrs1*^18^. While breeding lines ‘ICARDA4’ and ‘ICARDA9’ showed resistance to all isolates at seedling stage in one study^7^ and QTLs identified from them were projected at 383.9 Mb on the pseudomolecules Morex V 2.0 2019^18^, the identity of these resistances remain unknown.

A scald resistance QTL on the same chromosome arm as QTL-WAIYerong-3H (Fig. 2) was published by Coulter, *et al*.^17^. QTL qSUK7_3, contributed by the cultivar ‘Steptoe’ and identified from detached leaf assay phenotyping, was located 3 Mb away from QTL-WAIYerong-3H^17^ based on the pseudomolecules Morex V 2.0 2019, and 3 Mb away based on the pseudomolecules Morex 2017 (Fig. 2)^27^. The authors suggested this resistance to be different from *Rrs1* based on differential isolate reactions, but were unable to differentiate its map position from the *Rrs1* locus^17^. While it is difficult to draw conclusions from the relative distance between the QTL reported by Coulter, *et al*.^17^ and this study, further experiments are required to resolve whether the QTL-WAIYerong-3H is the same, or different to the locus identified in ‘Steptoe’ on chromosome 3H.

### Disease escape under field conditions

Our study illustrates the potentially confounding effects that relative maturity can have when phenotyping for disease resistance. The observed differences between genotypes may be conferred by both host resistance and mechanisms that lead to disease escape, such as plant maturity, plant height and canopy structure^8,15^. For example, Zhan, *et al*.^8^ postulated that later flowering time and taller plant height can physically slow the upward spread of splash-dispersed *R. commune* and contribute to disease escape. While disease escape traits are important, we sought to identify sources of resistance that are independent of relative maturity, as these QTL are more likely to be useful in breeding programs selecting for a constrained window of flowering time for their target environments.

In this study the positions of all four QTLs for relative maturity were co-located with known phenology QTLs. QTLRM-2H.1 was co-located with a QTL for heading date detected in a ‘TX9425’/‘Franklin’ population^28^ and this relative maturity allele was derived from ‘Franklin’. This QTL is close to another flowering-time gene, pseudo-response regulator *Ppd-H1*^29^, which is located 7 cM away on the Barley WGS Morex Assembly version 3^30^. QTLRM-2H.2 was located at the same position as another QTL for heading date detected by Nduulu, *et al*.^31^. QTLRM-5H and QTLRM-7H were located at the same positions as the flowering time loci *eam5* and *Eps-7S*, respectively^32^.

All of the loci we identified for relative maturity have also been implicated to be associated with resistance at seedling or adult plant stages to five biotrophic or necrotrophic pathogens. von Korff, *et al*.^33^ identified QTLs for scald, leaf rust and powdery mildew resistance at a position similar to that of *Ppd-H1*. QTLRM-2H.2 was located at the same position as a QTL for Fusarium head blight resistance detected by Nduulu, *et al*.^31^. The results of Nduulu, *et al*.^31^ also indicated that the QTLs for heading date and Fusarium head blight resistance were tightly linked rather than pleiotropic. A new gene conferring adult plant resistance to leaf rust, *Rph23*, was identified in the same ‘Yerong’/‘Franklin’ population used in this study on chromosome 7H, co-located with the QTL QTLRM-7H detected in this study^34^. Another QTL for stem rust resistance at the seedling stage was also mapped to the same position as QTLRM-7H from the same ‘Yerong’/‘Franklin’ population^35^.

A QTL for plant height was mapped to chromosome 2H at the same position as QTLRM-2H.2 in the ‘Yerong’/‘Franklin’ population by Xue, *et al*.^36^. Interestingly, the QTL for plant height identified in the ‘Yerong’/‘Franklin’ population is at the same position as a QTL for plant height in a ‘CM72’/‘Gairdner’ population^37^. Mature plant height was measured in only one of the experiments in this study (in 2017), and a correlation between mature plant height and scald resistance was not observed (data not shown), and the QTL identified for plant height did not co-locate with scald resistance QTL (Table 2).

### Modelling relative maturity together with disease resistance

The deviation from the regression of infection on important confounding traits has been employed previously as an effective phenotype for disease resistance^9,38^. Van Beuningen and Kohli^38^ in particular included linear functions for both heading date and plant height against Septoria tritici blotch (STB) infection in wheat. They aimed to calculate a phenotype that captured components of resistance that did not depend on those two traits, and represented a better approximation of genetic disease resistance from the experiments in their study. The residuals from a generalized linear model in which STB percentages were fitted to all escape related traits, including heading date, plant height, leaf spacing and leaf morphology, were used as the indicators of disease resistance to analyse the STB resistance in a set of wheat lines^39^. Chartrain, *et al*.^40^ identified a QTL for partial resistance to STB in wheat by using residuals from multiple regression on relative maturity and plant height as their phenotype. QTLs for spot blotch resistance in wheat were detected by using residuals when fitting disease severity as a dependent variable and plant height and days to heading as independent variables in multiple regression to exclude the effects of these traits^41^. Most of the reported uses of maturity regression residuals phenotype pertain to STB resistance in wheat. This could be due to the well-characterised relationship between STB infection rates and both flowering time and plant height^42^ and recognition that these confounding traits needed to be accounted for in ascertaining true genetic resistance for STB that would be useful for varietal improvement. In general terms, for phenotypes based on deviation from the regression of a correlated trait (like our DRSRRM measure of resistance) and the approaches described above, the most resistant lines are those with the largest negative values of residuals. This allows breeders to select resistant lines with desirable relative maturity and plant height^39,43^. Further, as noted by Van Beuningen and Kohli^38^, this approach is especially useful where disease resistance is evaluated in experiments at a single point in time, rather than at critical development stages for each genotype, especially in genetic material with large variation for confounding traits such as maturity.

In our study, a multiplicative mixed model was used to analyse the field data of scald resistance and relative maturity. When DRSRRM was utilised as a phenotype for QTL analysis, a major QTL was identified on chromosome 3H. This major QTL is co-located with a major QTL detected for scald resistance at the seedling stage in this study, QTL-WAIYerong-3H. When scald percentage of infected leaf is used as the phenotype for QTL analysis, a QTL for scald percentage on chromosome 2H were identified at the same position as QTLs for relative maturity; and the major QTL on chromosome 3H was also detected for scald percentage (Fig. 4). Our results show that by using DRSRRM as the disease resistance phenotype, the QTLs for disease resistance confounded by interactions with maturity were no longer significant in the QTL analysis.

In conclusion, a major QTL QTL-WAIYerong-3H providing scald resistance at both seedling and adult plant growth stages was identified. Results from this study indicate that this QTL-WAIYerong-3H is not *Rrs1*, as differential cultivar screening indicates the *Rrs1* (Rh3 type) allele from ‘Turk’ is not present in ‘Yerong’. The flanking markers for this QTL-WAIYerong-3H are located distantly (approx. 270 Mb) from the *Rrs1* (Rh4 type) locus based on the pseudomolecules Morex V 2.0 2019. To overcome the confounding effects of relative maturity on adult plant disease resistance, a bivariate approach was used to model the field data of scald resistance and relative maturity. The phenotype derived from the bivariate analysis (DRSRRM) is a more effective trait to detect disease resistance QTLs as it removes the confounding effects of relative maturity. The new QTL identified in this study is a useful resource for pyramiding different resistance genes (major or minor) in breeding programs.

## Methods

### Differential cultivar screening

The seedling scald resistance of different barley cultivars was tested against 11 *R. commune* isolates from the Wagga Wagga Agricultural Institute fungal isolate collection. These isolates were collected from southern New South Wales between 2013 and 2016 (Supplementary Table S2). The *R. commune* isolates were grown on lima bean agar (LBA) at 20 °C under 24 hour light condition^7^. After 2 to 3 weeks, spores were harvested with a scalpel blade and the spore solutions for spray inoculation were adjusted to 2 × 10^6^ spores ml^−1^ in distilled water using a haemocytometer. Seeds of cultivars were sown in 6-cm pots arrayed in a randomized complete block design consisting of 4 columns by 30 rows with three replicates of each genotype in each experiment for a total of 120 pots. A total of 40 cultivars were included in the differential cultivars screening, however, only the results for six key cultivars (‘Yerong’, ‘Franklin’, ‘Turk’, ‘Atlas’, ‘Atlas46’ and ‘Litmus’) were reported in this study (Table 1). Each cultivar by isolate combination was evaluated in at least two experiments. The responses of ‘Turk’ (*Rrs1*), ‘Atlas’ (*Rrs2*) and ‘Atlas46’ (both *Rrs1* and *Rrs2*) were compared with that of ‘Yerong’, while ‘Litmus’ was used as the susceptible check cultivar. The barley cultivars were uniformly sprayed with spore solutions at the 3-leaf-stage, and kept in a dark chamber for 48 hours at 18 °C with 100% humidity. The seedlings were scored 14 days post-inoculation with a scoring scale from 1 to 5 (1 = resistant and 5 = very susceptible) following the methods of Jackson and Webster^44^. For interpretation of the analysed isolate specific interactions, we define resistance as a BLUP score of up to 2.5 which is equivalent to the reactions of known major resistance genes *Rrs1* and *Rrs2*. We define moderate resistance as BLUPs in the range 2.5–3.5 and susceptible as scores of greater than 3.5. Conversely, an isolate is determined to be virulent if the BLUP was greater than 3.5.

### Plant material for QTL analysis

A DH population of 177 lines from a cross between the cultivars ‘Yerong’ and ‘Franklin’ was used in this study. Seeds for each genotype were obtained from the University of Tasmania. ‘Franklin’ is an Australian two-rowed malting quality cultivar, and ‘Yerong’ is an Australian six-rowed feed quality cultivar. The genetic linkage map of the population comprised 28 microsatellites and 196 diversity arrays technology (DArT) markers assembled by Li and Zhou^25^.

### Evaluation of seedling resistance to scald

Four different *R. commune* isolates, WAI2466, WAI2470, WAI2471 and WAI2473, were used in the seedling resistance screening in the glasshouse experiments (Supplementary Table S2). All four isolates were collected from southern New South Wales and are held in the Wagga Wagga Agricultural Institute fungal isolate collection (Supplementary Table S2). Inoculum preparation and inoculations were as outlined for the differential screening. Spores were diluted to 1 × 10^6^ spores ml^−1^ in distilled water using a haemocytometer. Each of the four isolates was tested in a separate experiment. For each experiment, the 177 DH lines as well as the parental cultivars ‘Franklin’ and ‘Yerong’ and an additional 21 check cultivars (for a total of 200 genotypes) were sown in 6-cm pots arrayed in a 24 column by 25 row randomized complete block design with three replicates of each genotype for a total of 600 pots. Each genotype by isolate combination was tested with three different experiments with three technical replicates per experiment. Seedlings were scored 14 days post-inoculation with a scoring system from 1 to 4 (1 = resistant and 4 = very susceptible) following the methods of Wallwork and Grcic^7^.

### Field screening

Field screening for scald resistance was conducted at Wagga Wagga Agricultural Institute (Wagga Wagga, New South Wales) in 2015, 2016 and 2017. All field trials were sown in May with a randomised complete block design with two replicates for each genotype. Each genotype was sown in 1.2 m rows with 0.4 m spacing between each row. The primary inoculum for *R. commune* infection was residual barley crop debris from the previous harvest. Overhead irrigation was used regularly to supplement rainfall throughout the growing season to enhance the development of disease. Experiments were subject to a strict weed control and crop nutrition regime to maximize yield potential. Assessment of disease was based on leaf symptoms using the percentage of infected leaf area^6^. Relative maturity at the time of disease assessment was determined using the Zadoks decimal score for plant development^45^. Final plant height was also measured at physiological maturity in the 2017 experiment.

### Statistical analysis

All data was analysed using the software package ASReml-R version 3^46^ in the R environment^47^. A linear mixed model following the approach of Gilmour, *et al*.^48^ was used to analyse the data for the differential cultivar screening experiments as follows (Model 1):

$${\boldsymbol{y}}={\boldsymbol{X}}\tau +{\boldsymbol{Z}}g+{\boldsymbol{Z}}u+e$$

where ***y*** is the *n* × 1 vector of the response variable (scald score) across *p* = 22 experiments with each of the 11 *R. commune* isolates tested in a separate experiment, and that experiment repeated once. *n* = 2640 for the differential cultivar screening experiments as only selected cultivars were included. ${\tau }$ is a $t\times 1$ vector of fixed effects for the overall mean scald score, corresponding to the $n\times t$ design matrix ***X*** The term *g* is the vector of genotypic random effects with associated design matrix ***Z*** used to model the genotype by experiment effects. The term ${\boldsymbol{u}}$ is the vector of random effects corresponding to the experimental design matrix $\,{\boldsymbol{Z}}$, which contains experiment-specific terms to capture extraneous variation for the experiment level blocking structures of replicate, row and column. The $n\times 1$ residual vector ${\boldsymbol{e}}$ was modelled for each experiment.

A model similar to Model 1 above was also used to model scald scores for the seedling inoculation experiments with $n=7200$ for the $p=12$ experiments conducted.

For the field screening experiments measuring scald resistance and relative maturity, each of the three field experiments was modelled separately using a bivariate approach as follows:

$$y=X\tau +Zu+e$$

where ${\boldsymbol{y}}$ is a vector of length $n=2\times 479$ containing stacked vectors for the two traits, $S$: scald resistance and $R$: relative maturity). ${\tau }$ is a vector of fixed effects including trait means and the trait by genotype effects for the design matrix ${\boldsymbol{X}}.$ The term ${u}$ is the vector of replicate, column and row effects for each trait corresponding to the experimental design structure ${\boldsymbol{Z}}$. The vector ${\boldsymbol{e}}$ of length $n$ containing the residuals of the two traits $S$ and $R$ was modelled with a separable autoregressive process of order one ($AR1\otimes AR1$) and an unstructured variance-covariance matrix between traits. This structure permits the fitting of a linear relationship at the residual level between the two traits. In all models, the significance of fixed effects was assessed using the techniques of Kenward and Roger^49^ and the significance of random effects other than ‘replicate’ was determined using log-likelihood ratio tests^50^.

The linear relationship between scald resistance and relative maturity was determined from the trait:genotype covariance modelling after the general approach of Van Beuningen and Kohli^38^ and the paired case-control study (Example 8.8) detailed in Butler, *et al*.^46^ as follows:

$${{\boldsymbol{e}}}_{S}={\beta }_{1}{{e}}_{R}+{\beta }_{0}$$

where the slope of the regression is calculated as:

$${\beta }_{1}=\frac{{\sigma }_{SR}}{{\sigma }_{S}^{2}}$$

and the intercept ${\beta }_{0}$ was determined from the overall experiment Best Linear Unbiased Predictions (BLUPs) for the two traits $S$ and $R$ from the bivariate mixed model. The difference (residual) between the BLUP from the mixed model for scald resistance of a genotype and the predicted value on the trend line was calculated. These values are referred to hereafter as the deviation from the regression of scald resistance on relative maturity (DRSRRM).

### QTL analysis and positioning of identified QTL on the barley physical map

The DRSRRM values for each individual field experiment were used as the phenotype to detect QTL for scald resistance adjusted for the relationship between scald resistance and relative maturity. For the phenotype analyses of scald percentage, relative maturity and plant height BLUPs were obtained from the model for each experiment. For the scald seedling-stage resistance phenotype, BLUPs were obtained from the seedling scald inoculation experiments model. Phenotypes were used for QTL analysis using the MapQTL6.0 software package^51^. QTLs were first analysed with interval mapping (IM). The closest marker to each QTL was selected as a cofactor for multiple QTL mapping (MQM). A logarithm of the odds (LOD) threshold value of 3 was used to identify QTL and QTL intervals.

The sequences of markers associated with the identified QTL for scald resistance and fine mapped *Rrs1* (Rh4 type)^12^ were used to perform BLAST searches by using the IPK Barley BLAST Server (http://webblast.ipk-gatersleben.de/barley). The barley pseudomolecules Morex V 2.0 2019 and pseudomolecules Morex 2017, were used for the BLASTn search. Default settings were used to do the BLASTn search and the best hit was used to decide the physical position of the detected QTL.

## Supplementary information

Supplementary Information.

## Data Availability

The datasets in the current study are available from the corresponding author on request.
