# Supplementary material for: Bivariate analysis of barley scald resistance with relative maturity reveals a new major QTL on chromosome 3H
Source: Sci Rep. 2019 Dec 30;9:20263. doi: 10.1038/s41598-019-56742-y (PMC6937342; doi:10.1038/s41598-019-56742-y)
Supplement: Supplementary file 1 — Supplementary Information. [file 41598_2019_56742_MOESM1_ESM.pdf]

# Bivariate analysis of barley scald resistance with relative maturity reveals a new major QTL on chromosome 3H

Xuechen Zhang[1], Ben Ovenden[1], Beverley A. Orchard[1], Meixue Zhou[2], Robert F. Park[3], Davinder Singh[3] and Andrew Milgate[1]*

[1]NSW Department of Primary Industries, Wagga Wagga Agricultural Institute, Wagga Wagga NSW 2650 Australia

[2]Tasmanian Institute of Agriculture, University of Tasmania, Private Bag 1375, Prospect, TAS 7250 Australia

[3]Plant Breeding Institute, The University of Sydney, Cobbitty, Private Bag 4011, Narellan, NSW 2567 Australia

* Corresponding author Email: andrew.milgate@dpi.nsw.gov.au Phone: +61 2 69381990

**Supplementary Table S1** Positions of marker sequences on 3H pseudomolecule (100 - 600 Mb) pseudomolecules Morex V 2.0 2019 and pseudomolecules Morex 2017

| QTL name | Closest markers | IPK pseudogenes 2019 | IPK pseudogenes 2017 |
|---|---|---|---|
| QTL-WAIYerong-3H | Bmag0006 | 120.3- 178.7 | 122.0 - 198.0 |
| qSUK7_3 | 1_1342-2_1129 | 181.5 - 467.4 | 201.2 - 508.8 |
| *Rrs1* (Rh4 type) | 11_0823 | 448.4 | 491.9 |
| Rrs1BC240 | MWG680 | 455.3 | 499.4 |
| QTLIA3H | YLM – MWG680 | 455.3 | 499.4 |
| qC147_3 | GBM1094-Bmag0112 | 418.1 - 556.6 | 458.0 |
| QTLD-3H-2015 | bPb-7872 | 503.4 | 503.4 |
| *Rrs4* | HVM36b–HVM60 | 523 | 576.6 |

**Supplementary Table S2** Origin of isolates of scald from the Wagga Wagga Agricultural Institute fungal isolate collection used for screening

| Isolate | Host Cultivar | Location | Collection date |
|---|---|---|---|
| WAI453 | Franklin | Wagga Wagga, NSW | 2013 |
| WAI1245 | Franklin | Wagga Wagga, NSW | 2013 |
| WAI2439 | Unknown | Downside, NSW | 2015 |
| WAI2463 | Buloke | Bogan Gate, NSW | 2015 |
| WAI2464 | Buloke | Bogan Gate, NSW | 2015 |
| WAI2466 | Buloke | Bogan Gate, NSW | 2015 |
| WAI2470 | Unknown | Mirrool, NSW | 2015 |
| WAI2471 | Unknown | Mirrool, NSW | 2015 |
| WAI2473 | Unknown | Mirrool, NSW | 2015 |
| WAI2636 | Buloke | Bogan Gate, NSW | 2015 |
| WAI2840 | Unknown | Finley, NSW | 2016 |
